# COMMUNITY-BASED PARTICIPATORY RESEARCH FILMMAKING WITH FORMERLY HOMELESS OLDER ADULTS

**DOI:** 10.1093/geroni/igz038.2078

**Published:** 2019-11-08

**Authors:** Victoria F Burns

**Affiliations:** University of Calgary, Calgary, Alberta, Canada

## Abstract

This methodological paper discusses the process of co-creating a documentary film with seven formerly homeless older adults, highlighting some of the tensions carrying out community-based participatory research (CBPR). This paper is part of a larger study that explored ‘finding home’ through a series of individual and group audio and video-recorded interviews (including walk and drive alongs) with seven adults (aged 50+) with diverse homeless histories. In addition to the main findings, participants shared their experience of filmmaking and CBPR. Findings revealed four main tensions: 1) openness of sharing stories versus privacy and anonymity; 2) balancing participation/engagement and over-burdening; 3) negotiating interpersonal conflict and community building; and 4) ethical issues surrounding copyright and ownership of the film. Ultimately, we advocate for more CBPR film projects, as they not only provide a rich contextualized window into people’s everyday lives but serve to advance the voices of marginalized populations beyond traditional academic circles.

